# Can scale cortisol concentration be quantified non-lethally in wild fish species?

**DOI:** 10.1093/conphys/coac081

**Published:** 2023-01-20

**Authors:** Emily K C Kennedy, David M Janz

**Affiliations:** Toxicology Undergraduate Program, University of Saskatchewan, 44 Campus Drive, Saskatoon, SK, S7K 5B3, Canada; Western College of Veterinary Medicine and Toxicology Centre, 52 Campus Drive, Saskatoon, SK, S7N 5B4, Canada

**Keywords:** steroid hormone, scales, physiological stress, glucocorticoid, biomonitoring

## Abstract

Cortisol, the primary glucocorticoid in fishes, is secreted into the bloodstream in response to stress. Circulating cortisol accumulates in scales, a durable calcified structure that can be easily sampled from many fish species. As such, the use of scale cortisol concentration (SCC) is currently being explored as a means of chronic stress biomonitoring in wild fishes. Scales serve an important role in fish physiology and thus the number of scales required for reliable cortisol analysis is a limiting factor in the non-lethal collection of such samples. To date, scale cortisol quantification has also only been performed non-lethally in captive fishes and due to differences in stress responsiveness SCCs likely differ in wild species. As such, this study aimed to (1) apply our fish scale processing and analysis protocol to wild fish species and (2) apply it to five north temperate fish species to provide information useful to future non-lethal scale sampling regimes. Cortisol was successfully measured in scales collected from wild northern pike (*Esox lucius*), walleye (*Sander vitreus*), whitefish (*Coregonus clupeaformis*), white sucker (*Catostomus commersonii*) and captive rainbow trout (*Oncorhynchus mykiss*). SCCs were significantly different between species and thus the sample mass required for reliable cortisol analysis differed as well. In addition to the size of the fish and the mass of their scales this is an important consideration for future scale cortisol analyses as these factors could make SCC an attainable non-lethal sample matrix in some species of fish but impractical in others.

## Teaser

Cortisol concentration in fish scales is proposed as a marker of long-term stress, but whether this can actually be quantified non-lethally in wild fishes is unclear. We determined scale cortisol concentration in five north temperate fish species and concluded that large-bodied fishes with large scales are suitable for this purpose.

## Introduction

Fish wellbeing is an important consideration for aquaculture, research ethics and a healthy ecosystem. As human populations continue to expand, the frequency and duration of stressors applied to fishes in both wild and captive settings is on the rise. Fishes exposed to stressful stimuli initiate a stress response mediated by the hypothalamic–pituitary–inter-renal (HPI) axis that results in the production of cortisol followed by its distribution via the bloodstream (Wendelaar Bonga, 1997; Koakoski *et al.,* 2012). Once circulating in the blood, cortisol acts to increase energy mobilization for use in homeostatic restoration (Janssens and Waterman, 1988; Koakoski *et al.,* 2012; Lawrence *et al.,* 2019). This is advantageous in response to predation and other acute stressors but when stimulated for long periods of time, the stress response may rob other physiological processes of the energy needed to carry out important tasks (Wendelaar Bonga, 1997). In addition, cortisol receptors distributed throughout the organism provide additional opportunity for adverse effects (Groeneweg *et al.,* 2012; Sadoul and Vijayan, 2016). Fishes experiencing long-term stress are therefore at higher risk of a wide variety of physiological alterations including reduced immunity (Small and Bilodeau, 2005; Tort, 2011), problems with osmoregulation (Laiz-carrión *et al.,* 2003), stunted growth (Pickering, 1993) and reproductive inhibition (Schreck *et al.,* 2001). Due to increased energetic and temporal costs of reproduction, a variety of stress-induced adverse responses including hormonal alterations (Pickering *et al.,* 1987) and reduced gamete quality (Campbell *et al.,* 1994; Valdebenito *et al.,* 2013) have been reported. The consequences of long-term stress could thus lead to population level effects in fishes, emphasizing the need for regular monitoring techniques that minimize additional stress.

Integumentary structures such as hair and feathers have been shown to incorporate and store cortisol and are thus used in the assessment of chronic stress in mammals and birds. To provide similar information in fishes the past decade has seen numerous advancements in the use of fish scale cortisol concentration (SCC) in the assessment of chronic stress. Although specific mechanisms of scale cortisol deposition are still unknown they have been shown to retain cortisol for a longer period of time than other media such as blood or mucus (Aerts *et al.,* 2015; Laberge *et al.,* 2019). As such, increases in SCC have now been associated with overstocking and other changes in aquaculture practices (Hanke *et al.,* 2020), fin injuries (Weirup *et al.,* 2021) and increases in water temperature (Hanke *et al.,* 2019) demonstrating its potential as a diagnostic tool. As has been proposed by several groups, the ultimate goal in the development of these methods is conservation (Aerts *et al.,* 2015; Carbajal *et al.,* 2019b; Roque d’orbcastel *et al.,* 2021). Therefore, the collection of scales for hormone quantification purposes should ideally be performed non-lethally; however, the practicality of this condition in wild fishes has yet to be assessed. While some studies have been performed in wild-caught species, none of said studies were carried out non-lethally (Carbajal *et al.,* 2019b; Culbert *et al.,* 2021; Roque d’orbcastel *et al.,* 2021). In addition, while some groups have successfully collected scales non-lethally these studies were performed in-lab (Aerts *et al.,* 2015; Hanke *et al.,* 2019). Differences in stress responsiveness between wild and captive species can differ significantly (Sopinka *et al.,* 2016). This suggests SCCs will also be different between wild and captive species, which will contribute to the sample mass required from each species. As scale sample mass is a limiting factor in non-lethal scale sampling for cortisol quantification, preliminary assessments of these techniques in both wild and captive fish is required. Additionally, the use of SCC as a biomonitoring tool will likely be achievable only via comparison between populations of the same species collected at different times or from different bodies of water, necessitating the quantification of SCC in as many species, at as many times of year and in as many locations as possible. To this end our study served to ensure our previously validated scale sample processing protocol was applicable to wild species and generate additional information on sample masses required from each of the five species of fish used in this study to guide future studies.

## Methods

Five fish species were sampled for scales: three large scaled fish species, adult whitefish (*Coregonus clupeaformis*, *n* = 10), adult white sucker (*Catostomus commersonii*, *n* = 12) and adult northern pike (*Esox lucius*, *n* = 10); one medium scaled fish species, adult walleye (*Sander vitreus*, *n* = 10); and one small scaled fish species, juvenile rainbow trout (*Oncorhynchus mykiss, n* = 8). Fish were deemed large scaled if scales weighed more than 5 mg, medium scaled if scales weighed 1–5 mg and small scaled if scales weighed less than 1 mg. An average scale length and height generated from measurements of five scales per species were also recorded but scale size classification was determined only by scale mass. All fish were collected in late July, whitefish, northern pike and walleye were collected from Blackstrap Lake, SK, white sucker were collected from Lake Diefenbaker, SK, and rainbow trout were from a lab colony at the University of Saskatchewan. All scale samples were collected opportunistically during field collections and laboratory research performed by other researchers, which were conducted using Animal Use Protocols approved by the Animal Research Ethics Board of the University of Saskatchewan Animal Use and Care Committee. Northern pike average mass and length were 1.56 kg and 57.6 cm, respectively, for walleye these values were 1.47 kg and 18.5 cm, respectively, and for rainbow trout average mass was 0.02 kg and average length was 11.2 cm. Lengths and masses were recorded when available; however, scales used in this study were gathered from fish collected by other research groups and thus this information was unavailable for white sucker and whitefish. Nevertheless, these were all adult fish similar in size to the northern pike used in this study. SCC was determined by adapting methods validated in our previous study conducted using goldfish (*Carassius auratus*) from lab-stocks (Kennedy and Janz, 2022).

### Sample collection and storage

All whitefish, northern pike, walleye and rainbow trout scale samples were collected post-mortem. With the exclusion of rainbow trout, fish were caught via gill netting, euthanized with a sharp blow to the head and sampled for scales approximately four hours post-mortem. Three white sucker samples were collected post-mortem as described above and nine were sampled from live specimens. Live white sucker were restrained for ~1 min without anesthesia while scales were plucked using forceps and then released. Approximately 5–7 scales were sampled from four regions of the body on one side of the fish to obtain a representative whole-body scale sample comparable to the rainbow trout used in this study as well as the goldfish in our previous study as it was necessary to descale the entire body of these fish to obtain sufficient scale mass. Unlike the other species of fish, juvenile rainbow trout were euthanized via anesthetic overdose. As Laberge et al. (2019) demonstrated, cortisol generated by acute stressors such as that induced during sampling will likely not be incorporated into the scale due to the lag in transfer of cortisol from blood to scale and thus differences between live-sampled and post-mortem sampled fish were not expected. All scales were stored at −20°C until processing for a maximum of one week. Cortisol has been shown to be highly stable in hair samples for lengthy periods of time and while this has yet to be assessed in scales it is unlikely that significant cortisol breakdown occurred during storage.

### Removal of surface contamination

Scale samples can be contaminated with other hormone-containing media such as mucus and blood. To remove contaminants and ensure accuracy of internal SCC, scale samples were briefly washed three times with methanol as it is capable of removing lipophilic contaminants such as steroid hormones (Kroshko *et al.,* 2017). This involved placing samples from fish with large- and medium-sized scales (whitefish, northern pike, walleye and white sucker) in a 15-ml falcon tube with 12 ml of methanol and small scale samples (from rainbow trout) into a 5-ml falcon tube with 4 ml of methanol and gently vortexing for 2.5 min. Between each wash, methanol was decanted, scales were blotted dry and any visible debris (skin, etc.) was removed using forceps. Wash tubes were rinsed between each wash and a fresh aliquot of methanol was used for each successive wash. After three washes, scales were placed in a filter paper-lined petri dish with the lids off-set for air flow and dried at room temperature for 24 h prior to further processing.

Previous studies determining SCC used ultrapure water (Aerts *et al.,* 2015) or isopropanol (Carbajal *et al.,* 2018, 2019a,b) for washing purposes. However, Carbajal et al. (2018) suggested that water may be capable of penetrating the scale and removing internal scale hormone and that isopropanol was perhaps not sufficiently aggressive to remove of external hormone contributed by blood or mucus. As mucus contains many compounds insoluble in water such as fatty acids, methanol, a nonpolar solvent previously validated both in our earlier work conducted in goldfish and in the decontamination of hair samples was chosen (Kroshko *et al.,* 2017; Kennedy and Janz, 2022). Our previous study in goldfish demonstrated that a minimum of two and a maximum of three washes with methanol was recommended for the removal of external cortisol, DHEA and cortisone from the scale (Kennedy and Janz, 2022). However, many species of fish used in this study had scales of a greater thickness and larger surface area than goldfish. Thus, to ensure these methods were appropriate for the larger-scaled, wild-caught species used in this study we re-validated this wash protocol in this study. To do so, a subset of five whitefish scale samples was washed one to five times and the cortisol concentration of all five wash solutions as well as their matching scale samples were determined independently. This method of wash solvent efficacy validation has been previously employed by groups exploring the use of both scale and hair hormone quantification for chronic stress assessments (Kroshko *et al.,* 2017; Carbajal *et al.,* 2018). Whitefish scales were chosen as they were representative of a larger wild-caught fish species and they were in high abundance at the time of method validation. For all fish species, scales were weighed prior to washing as well as post washing and drying to determine the required mass of sample necessary for reliable cortisol analysis. Ten individual scales were also weighed post washing to estimate the approximate number of scales needed from each fish species to allow for adequate dry sample mass.

### Grinding

Washed and dried scales were minced with scissors in a glass vial prior to being ground to a fine powder using a Retsch ball mixer mill. Samples were ground in a 10-ml grinding jar with a 12-mm stainless steel grinding ball for 0.045 s per mg of scale at 30 Hz. Sample masses of 5, 10, 25 and 50 mg of scale were analyzed for SCC to determine a preliminary mass required for analysis. This was again done using whitefish scales as they were abundant at the time of method validation; however, the minimum mass required for analyses for each species was re-evaluated post-cortisol quantification as SCC differed markedly among species.

### Cortisol extraction and quantification

Methanol was added to each powdered scale sample (0.2 ml methanol/mg scale) and vortexed briefly for 15 s. Tubes were then placed in a rotator and left for 18 h to extract at room temperature. Extracted samples were centrifuged for 15 min at 4500 rpm and 20°C. Extracts were collected in 5 ml borosilicate glass tubes and dried at 38°C under a gentle stream of nitrogen gas. Methanol (0.2 ml/mg) was added back to the powdered samples and vortexed for 40 s, then centrifuged, collected and evaporated as above. These steps were repeated twice for a total of three collections. To concentrate extracted cortisol at the bottom of each tube the sides were rinsed four times with decreasing volumes of methanol (1 ml, 0.4 ml, 0.2 ml, 0.15 ml). Between each rinse extracts were dried at 38°C under nitrogen gas.

Extracts were reconstituted in 200 μl of extraction buffer supplied with the cortisol enzyme immunoassay (EIA) kit (EA65, Oxford Biomedical, East Lansing, MI). Tubes were sealed with parafilm and vortexed gently for 10 s, then wrapped in tin foil and incubated for 12 h at 4°C. After 12 h, tubes were gently vortexed for 15 s and the reconstituted sample was transferred into a 0.6-ml Eppendorf tube. Finally, samples were centrifuged at 4500 rpm and 20°C for 5 min to remove any trace scale residue and the supernatant was collected. Samples were run in triplicate following the EIA kit protocol using a Molecular Devices Spectra Max 190 microplate spectrophotometer.

Extracts from four samples were pooled for intra-assay variation (*n* = 5) and inter-assay variation (*n* = 10), determined using percent coefficient of variation (%CV, SD/mean × 100%). Parallelism between extracted samples and the kit standard curve was determined using a serial dilution of the pooled extract run in triplicate. Intra-assay and inter-assay variation were 5.9% and 12%, respectively. Parallelism was observed between the standard curve and serially diluted extract. This was determined by comparing the hill slope of the curves plotted using a four-parameter log-logistic model; if the hill slope did not differ significantly between the two curves they were deemed parallel. The limit of detection of the EIA was 0.005 ng/ml.

### Determination of mass required for analysis

The seven cortisol standards provided by the EIA kit used in this study range from 0.005–50 ng/ml with the linear range of the standard curve encompassing standards three to five that range from 0.1–2 ng/ml. Standard three (0.1 ng/ml) was thus chosen as the minimum desirable concentration obtained from a scale sample for reliable cortisol analysis using this EIA kit. Using the average SCC for each species and the concentration of standard three as a minimum desirable extract concentration a minimum scale mass required for analysis was determined for each species (Table 1).

**Table 1:** Scale sampling guide for SCC analysis in five fish species using ELISA

Species	Dry scale mass required (mg)	Corresponding wet scale mass (mg)	Corresponding # of scales	Average scale length (mm)	Average scale height (mm)
Northern pike	3.20	5.08	1	10.2	6.9
Rainbow trout	34.6	308	35	1.2	1.3
Walleye	23.2	36.3	7	7.4	7.8
Whitefish	37.4	68.0	5	9.6	10.1
White sucker	5.60	9.93	1	13.4	9.2

### Statistical Analyses

Normality and homoscedasticity of the SCC data from all five species were tested using a Shapiro–Wilk and Bartlett’s test, respectively and all data sets failed assumptions for parametric statistical analyses. Thus, to detect differences in SCC among fish species a Kruskal-Wallis test followed by Dunn’s test was used, and differences were determined to be significant at p < 0.05.

## Results

### SCC protocol validation

**Figure 1:**
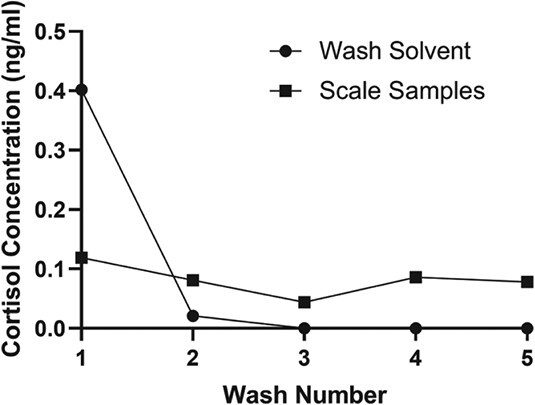
Comparison of cortisol concentrations in wash solvents and matching scale samples washed 1–5 times with methanol independently.

Cortisol concentration decreased from 0.40 ng/ml in the first wash solution to 0.02 ng/ml in the second wash solution to below the detection limit of the EIA (0.005 ng/ml) for all subsequent washes (Fig 1). We therefore selected three washes with methanol for subsequent SCC analyses. Additionally, cortisol concentrations in the scale sample extracts ranged from 0.044 to 0.12 ng/ml and did not show any decline with the increasing number of washes indicating that internal cortisol was not being leached from the whitefish scales for up to five washes. This was additionally confirmed by our previous study in goldfish (Kennedy and Janz, 2022) as scales from this study, washed using the same protocol, had similar cortisol concentrations as a study by Laberge et al. (2019) that used ultrapure water to wash scales of the same species. This suggests that the choice of wash solvent does not appreciably affect the internal SCC (Kennedy and Janz, 2022).

Extracts from 5 and 10 mg of dry powdered whitefish scale did not contain sufficient cortisol to be detected using the EIA. The extracts from 25 mg and 50 mg of scale contained 0.009 ng/ml and 0.078 ng/ml, respectively, which were both on the low end of the cortisol standard curve. Thus, we determined that 100 mg of dry powdered scale was required to ensure the extract had a cortisol concentration high enough for reliable detection using this EIA kit in all species of fish.

The dry mass of individual scales varied between species and was <1 mg for rainbow trout, 3.7 mg for walleye, 6.7 mg for northern pike, 8.9 mg for whitefish and 19 mg for white sucker, as did scale sample moisture content. Scale moisture contents for walleye, northern pike, whitefish and white sucker were relatively consistent ranging from 35.5% to 44.8%; however, this value was greater in rainbow trout (88.8%) likely a result of increased mucus. Thus, to obtain a scale extract of at least 0.1 ng/ml, the corresponding dry mass and thus the number of scales also varied between species (Table 1). Scale length and height measurements are also provided in Table 1 as well as a recommended wet sample mass to facilitate future scale sampling. Due to the small quantity of scales available for collection from individual rainbow trout, the scales collected from two fish were pooled to meet the preliminary scale mass requirements, decreasing the likelihood of sampling this species non-lethally in future monitoring efforts.

### Scale cortisol concentrations

**Figure 2:**
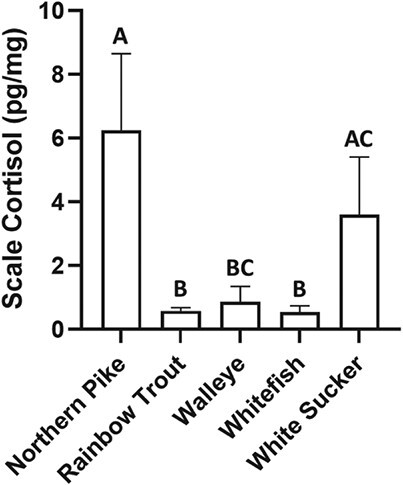
Comparison of SCCs in five north temperate fish species. Data are shown as mean ± 95% CI of *n* = 8–12 fish and letters are used to denote significant differences (*P* < 0.05).

SCC in all five species are presented in Fig. 2. SCC in northern pike was significantly greater than rainbow trout (*P* = 0.0009), walleye (*P* = 0.0103) and whitefish (*P* = 0.0001). In white sucker, SCC was significantly greater than rainbow trout (*P* = 0.0451) and whitefish (*P* = 0.0104). No other comparisons were deemed statistically significant (*P* > 0.05).

## Discussion

Integumentary structures such as scales provide a potential means of evaluating the cumulative activity of the physiological stress response in fish over relatively long periods of time. Due to the differences in SCC among fish species reported here and in other studies, the quantity of dry scale mass required to reliably detect SCC following the methods described herein will vary significantly. In the case of northern pike and white sucker SCC reported here, SCC quantification in these species could be accomplished with less than five scales. Although it cannot be fully confirmed by the results of this study, successful non-lethal scale sampling has been achieved in lab using a greater number of scales in fish of a smaller size suggesting that non-lethal sampling will also be plausible in the species used in this study (Aerts *et al.,* 2015). This also suggests that the implementation of such methods in biomonitoring regimes will be more easily accomplished in some species as well as at certain life stages, determined by the SCC, the size of the fish scales and the size of the fish. As scales provide protection to the organism both from mechanical injury and biological pathogens it is important to consider the number of scales being removed relative to the size of the fish.

The methods for fish scale processing and analysis described in this study were adapted from our previous work in goldfish (Kennedy and Janz, 2022). However, as certain fish species analyzed in this study had much larger scales than goldfish (i.e. whitefish, white sucker and pike) these methods were re-validated in whitefish scales, a representative large-scaled, wild-caught species. The degree of external scale contamination also may have differed between wild-caught and captive species, necessitating this additional validation. While three washes with methanol appeared to be sufficient in the removal of external cortisol as was the case with goldfish scales, the amount of dry mass required for reliable cortisol quantification was two-fold greater in certain species. Compared to our prior study in goldfish, scales collected from whitefish and rainbow trout contained lesser amounts of cortisol and will require between 50 and 100 mg of dry scale for reliable SCC determination (Kennedy and Janz, 2022). This is the main limiting factor when attempting to obtain scale samples non-lethally and the reason why euthanasia cannot be avoided when sampling some species of fish. Additionally, pooling scales from multiple animals may be necessary, as was the case with the juvenile rainbow trout in this study.

SCC was greatest in northern pike and white sucker with walleye, rainbow trout and whitefish having similarly low concentrations. White sucker circulating cortisol has been reported to be as high as 200–250 ng/ml (McMaster *et al.,* 1994). By contrast, northern pike circulating cortisol has been reported to be as low as 5 ng/ml; however, these results were from a study conducted during the winter (Louison *et al.,* 2017). Northern pike are a desirable angling fish suggesting that the time of collection for this study represented a highly stressful period for this species. Pullen et al. (2017) demonstrated that a simulated capture and release event could raise circulating cortisol in northern pike up to 385 times their recorded baseline perhaps explaining the high SCC reported in this study. However, as we cannot confirm the state of stress in the fish used in this study the SCC reported here will likely be more useful for intraspecies comparisons in future studies. This is currently possible only in rainbow trout, which had similar SCC as those reported by Carbajal et al. (2019a), Weirup et al. (2021) and in our recent study (Kennedy and Janz, *in review*). The remaining species had SCC similar to those reported in other species such as wild Catalan chub *(Squalius laietanus)* and two species of tuna *(Katsuwonus pelamis* and *Thunnus albacares)* with the exception of northern pike, which had one of the highest SCC values reported to date (Carbajal *et al.,* 2019b; Roque d’orbcastel *et al.,* 2021).

Intermittent sampling over long time periods has been achieved in mammals using hair (Macbeth *et al.,* 2010; Bechshøft *et al.,* 2012) and in birds using feathers (Bortolotti *et al.,* 2009) as non-lethal measures of long-term stress. Associations between hair cortisol concentration and other physiological parameters have also been observed in mammals, suggesting that changes in SCC over long periods of time may be useful in assessing the effects of current threats to fishes such as climate change and pollution as well as overstocking in aquaculture and lab stocks (Bechshøft *et al.,* 2013, 2015; Sergiel *et al.,* 2017; Hanke *et al.,* 2020). Beaudin et al. (2010) determined mercury concentrations in serial sections of fish scales allowing for a chronological assessment of mercury exposure. This suggests that fish scales could be used in the assessment of stress axis activity in association with toxicant exposure and potentially other stressors. Further investigation of scale sampling techniques may provide a practical means of assisting in the conservation of wild fish species and prove useful in assessing the welfare of aquaculture populations.

Some have argued that it is incorrect to consider scale sampling as non-invasive or non-stressful due to the nature of sample collection, and success with less-invasive means of sampling cortisol concentrations have indeed been reported (Sadoul and Geffroy, 2019). For example, Cao et al. (2017) evaluated stress in farmed Atlantic salmon (*Salmo salar*) using fecal cortisol concentrations. Other groups have reached similar goals using the surrounding water environment (Fanouraki *et al.,* 2008; Gabor and Contreras, 2012) and mucus (De Mercado *et al.,* 2018) cortisol concentrations. However, while these methods are less invasive than scale sampling, similar to blood these media only provide insight into short temporal snapshots of physiological stress in fish rather than longer-term stress (Carbajal *et al.,* 2019a). Scales are unique in this regard as their turnover rate is lengthy in comparison with other sampling media, as indicated by their use in fish ageing (Ilieş *et al.,* 2014). Although the cortisol retention is likely much less permanent than hair as demonstrated by Laberge et al. (2019), blood supplied to the scale appears to result in the deposition and retention of steroid hormones within the collagen matrix of the scale for a longer period of time than any other media (Aerts *et al.,* 2015; Hanke *et al.,* 2019, 2020; Laberge *et al.,* 2019; Carbajal *et al.,* 2019b). The non-lethal sampling of fish scales has been achieved multiple times in the laboratory using similar proportions of scale to those proposed in this study, suggesting that this is indeed plausible in wild species (Aerts *et al.,* 2015; Hanke *et al.,* 2019). Additional support for this comes from a study conducted by Baker et al. (2004) during which they sampled small amounts of muscle tissue from northern pike for mercury analysis, a sample process similar to scale collection, without affecting survival of the fish for up to a year post-sampling. A study performed by Sire (1989) in a species of cichlid fish (*Hemichromis bimaculatus*) also demonstrated that the small wound induced by scale removal is healed within a very short period of time (3–6 h), limiting the opportunity for infection and additional harm.

## Conclusion

The development of non-lethal sampling methods are essential tools in the successful conservation of wild species. This study provides useful information for future biomonitoring regimes and suggests that measuring SCC will likely be plausible via non-lethal methods in certain fish species, and therefore has potential as a tool for conservation efforts. This will depend on multiple contributing factors such as actual SCC, scale size and body size. Further development of scale sampling and processing protocols may provide researchers and managers with a means of evaluating long-term stress in many species of fish, potentially allowing for the mitigation of stress-inducing factors and the protection of economically, ecologically and culturally important fish populations.

## Funding

This research was supported by a Natural Sciences and Engineering Research Council of Canada (NSERC) Discovery Grant to DMJ [RGPIN-2016-05131].

## Data availability

The data underlying this article are available in the article and in its online supplementary material.
